# The sex‐specific prognostic utility of sarcopenia in cirrhosis

**DOI:** 10.1002/jcsm.13059

**Published:** 2022-08-09

**Authors:** Ryan Lowe, Penelope Hey, Marie Sinclair

**Affiliations:** ^1^ University of Melbourne Melbourne Victoria Australia; ^2^ Northern Health Epping Victoria Australia; ^3^ Liver Transplant Unit Austin Health Heidelberg Victoria Australia

**Keywords:** Sarcopenia, Cirrhosis, Sex, Frailty, Mortality, Prognosis

## Abstract

Sarcopenia is an increasingly recognized complication of cirrhosis that is associated with morbidity and mortality. Differences in the prevalence and prognosis of sarcopenia between men and women have been reported in other patient groups, but there is insufficient understanding of how sex impacts the prognostic value of sarcopenia in cirrhosis. A search of MEDLINE and Embase was conducted from earliest entries to April 2021. Studies were included if they examined sex‐stratified mortality impact of reduced muscle function or mass in outpatient populations with cirrhosis. We identified 700 studies of which 6 were deemed relevant for inclusion in this narrative review. Studies of interest were heterogeneous, precluding pooling of data and making interpretation of the literature challenging. Muscle mass was assessed in five studies (*n* = 2566, 1730 men, 836 women) and was reduced in 36–50% of men and 24–43% of women. All five studies found that reduced muscle mass determined by computed tomography, dual‐energy X‐ray absorptiometry, and bioelectrical impedance analysis was associated with increased mortality in men. Of these, two studies identified a corresponding relationship in women; reduced muscle mass defined by computed tomography was associated with increased mortality [hazard ratio (HR) 2.82, *P* = 0.001], while increasing muscle mass by bioelectrical impedance analysis likewise conferred a survival benefit (HR 0.45, *P* = 0.0016). Only one study assessed the relationship of muscle function with sex‐stratified mortality (*n* = 1405, 827 men, 578 women), concluding that reduced muscle function predicted mortality in both men and women (HR 1.65, *P* < 0.001 and HR 1.54, *P* < 0.001, respectively). Reduced muscle mass in cirrhosis is consistently associated with mortality in men, but lack of sex‐stratification of mortality analyses limits the ability to make strong conclusions about the impact of sarcopenia specifically in women, with even fewer data available for analysing muscle function. Improved understanding of the sex‐specific impacts of sarcopenia may help address patient deterioration and mortality while awaiting liver transplantation and allow for early intervention to mitigate mortality risk. Large, multicentre studies with adequate female participation and sex‐stratified mortality analyses are warranted.

## Introduction

Cirrhosis is a global disease with significant morbidity and mortality. Over 1.3 million deaths resulted from cirrhosis in 2017, accounting for 2.4% of deaths worldwide.^1^ The classically recognized features of jaundice, ascites, oesophageal varices, and hepatic encephalopathy are well‐understood clinical complications of cirrhosis, with sarcopenia only recently capturing attention. There are multiple pathways that are thought to contribute to the development of sarcopenia in cirrhosis, including portal hypertension, systemic inflammation, changes in hepatic metabolism, and hormonal alterations.^2^

Expert consensus from the European Working Group on Sarcopenia in Older People currently requires evidence of impaired muscle function to make a presumptive diagnosis of sarcopenia before confirming either low muscle mass or low muscle quality.^3^ Evidence suggests that low muscle mass alone as measured by cross‐sectional imaging is present in up to 70% of patients with cirrhosis and carries significant contributions to morbidity and mortality.^4^ Thus, sarcopenia has been considered as an attractive prognostic tool that might aid management decisions, including patient prioritization for liver transplantation.

While sarcopenia has been validated as a prognostic marker in cirrhosis, few studies directly address the influence that sex may have on muscle parameters beyond the intermittent use of sex‐specific definitions of reduced muscle function, mass, or quality. Studies are almost invariably performed in male‐dominated cohorts, which is problematic as results cannot be generalized because muscle homeostasis is known to be under the control of multiple factors, some of which are sex specific.^5^ Studies in geriatric populations have found that while muscle loss occurs less frequently in ageing women, they experience significantly higher mortality compared with their male counterparts because of reduced muscle mass.^6^ Given that only a minority of studies have attempted to verify mortality associations in both men and women, further understanding is required in this area.

This review examines the current literature to understand the relationship between sex and the prognostic significance of sarcopenia in cirrhosis.

## Methods

### Literature sources and search strategy

A comprehensive MEDLINE and Embase electronic database search was performed from earliest entries to April 2021 using the search terms including ‘cirrhosis’, ‘liver fibrosis’, and ‘liver failure’ with ‘frailty’, ‘sarcopenia’, and ‘muscular atrophy’, as well as ‘mortality’, ‘prognosis’, ‘survival’, and ‘death’. Medical Subject Headings, Boolean operators, and truncations were also utilized throughout the strategy (Supporting Information, *Appendix* *S1*). Articles were further identified via directly searching for authors known to be experts in the field.

### Inclusion and exclusion criteria

Inclusion criteria included studies that recruited patients with cirrhosis over the age of 18 from an outpatient population. Differing definitions and methods used to diagnose sarcopenia prohibited comparison between studies. Only studies that recruited both men and women were considered for inclusion. Studies were included if they assessed the impact of muscle function or mass on mortality in both men and women with cirrhosis before or without liver transplantation.

Exclusion was deemed appropriate when studies recruited from an acutely ill or hospitalized population or demonstrated an isolated interest in patients with hepatocellular carcinoma. Studies that examined changes in muscle parameters over time and investigated biomarkers, nutritional markers, or exercise physiology were excluded. Studies that included the same study cohort as reported in other studies were excluded as were those studies not published in English and where full text was not available.

## Results

**Figure 1:**
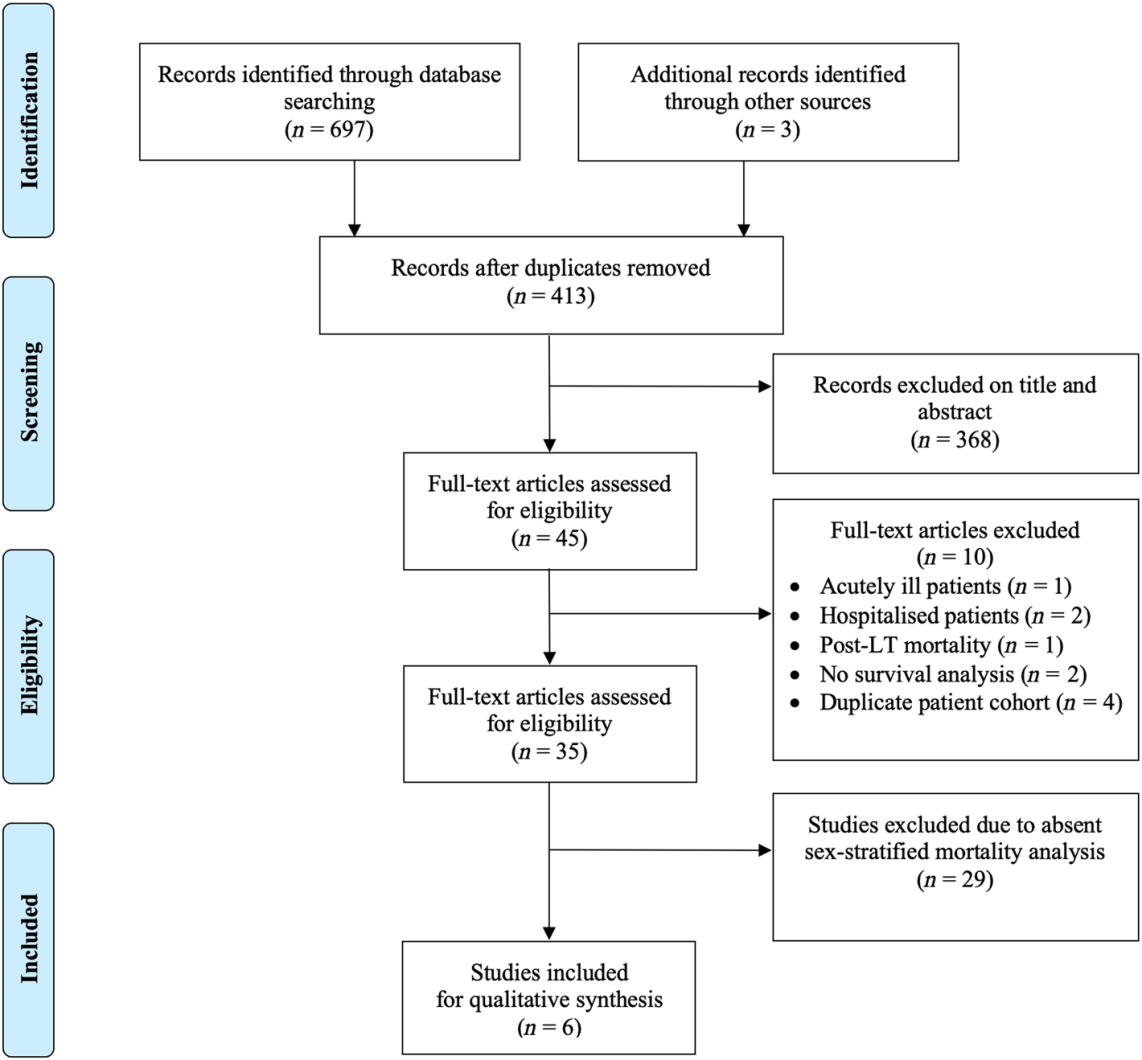
Study selection process. LT, liver transplant.

The initial search identified 380 and 317 citations from MEDLINE and Embase, respectively. A further three citations were identified by searching expert authors. From the total of 700 citations, 287 were removed as duplicates. Screening by a single reviewer (R. L.) based on title and abstract highlighted 45 citations that remained of interest. Ten papers were excluded following a review of full text, leaving 35 studies that used a sex‐stratified diagnosis of sarcopenia. A further 29 were excluded due to lack of sex‐specific mortality analyses; thus, 6 studies of interest were included in this review (*Figure* 1).

These six identified manuscripts captured 3971 patients, 2557 (64%) of whom were men and 1414 (36%) of whom were women. Most studies were conducted in North America, which also contributed the largest patient cohort. Patients awaiting transplantation predominated the populations of included studies. Liver disease aetiology differed with sex and location of the study. Time to follow‐up across the studies ranged from 8.8 months to 3.2 years.

Muscle mass was analysed in five studies (*n* = 2566, 1730 men, 836 women) that took a sex‐stratified approach to mortality assessment (*Table* 1). In each of these studies, reduced muscle mass was associated with mortality in men, whereas only two studies identified a significant relationship between muscle parameters and mortality in women. Muscle mass was commonly assessed by obtaining the skeletal muscle index (SMI) by computed tomography (CT) at the level of the third lumbar vertebrae (L3). Using this method, low muscle mass was present in 42–50% of men and 24–33% of women. When viewed as a dichotomous variable, one study found that L3 SMI was significantly associated with mortality in both men and women.^7^ A continuous analysis of muscle mass found that each per‐unit increase in L3 SMI was significantly associated with survival in men alone.^8^ Similarly, cross‐sectional analysis of the paraspinal muscle at the L3 vertebral level was found to significantly predict mortality in men, but not in women.^9^ Muscle mass assessed by dual‐energy X‐ray absorptiometry classified 49% of men and 43% of women as having low muscle mass by appendicular SMI, with only men demonstrating a significant survival benefit with increasing appendicular SMI.^10^ Bioelectrical impedance analysis determined muscle mass was significantly associated with mortality in both men and women.^11^ Muscle mass determined by this method was found to be reduced in 36% and 28% of men and women, respectively.

**Table 1:** Sex‐stratified association between muscle mass and mortality in cirrhosis

Authors (year) Region Sarcopenia measure	Participants	Status	Disease severity (MELD)	Disease aetiology	Definition of muscle loss	Muscle loss prevalence	Impact on mortality	Time to follow‐up
Carey *et al*. (2017)^7^ North America L3 SMI by CT	M: 277	Awaiting LT	14.4 (median)	HCV (54%) Alcohol (18%) NASH (11%)	<50.0 cm^2^/m^2^	50%	HR 1.7 (*P* = 0.03) for waitlist mortality on multivariate analysis in men with low SMI	8.8 months (median)
	W: 119		14.7 (median)	HCV (33%) AIH/PBC/PSC (24%) NASH (15%)	<39.0 cm^2^/m^2^	33%	HR 2.82 (*P* = 0.001) for waitlist mortality on multivariate analysis in women with low SMI	
Ebadi *et al*. (2018)^8^ North America L3 SMI by CT	M: 456	Awaiting LT	14 (average)	HCV (44%) Alcohol (25%) NASH (20%)	<50.0 cm^2^/m^2^ for men	42%	HR 0.98 (*P* = 0.02) for mortality on multivariate analysis per‐unit increase in SMI in men	20 months (average)
	W: 221		15 (average)	HCV (29%) NASH (29%) AIH/PBC/PSC (20%)	<39.0 cm^2^/m^2^ for women	24%	HR 0.99 (*P* = 0.24) for mortality on univariate analysis per‐unit increase in SMI in women	
Engelmann *et al*. (2018)^9^ Europe L3/L4 PSMI by CT	M: 561	Awaiting LT	15.8 (average)	Alcohol (62%) Other (22%) HBV/HCV (10%)	—	—	HR 0.933 (*P* = 0.005) for mortality on multivariate analysis in men per‐unit increase in PSMI	12 months pre‐transplant
	W: 234						HR 0.937 (*P* = 0.161) for mortality on multivariate analysis in women per‐unit increase in PSMI	
Eriksen *et al*. (2021)^10^ Europe ASMI by DXA	M: 231	Not awaiting LT	14	HCV (35%) ETOH (17%) NASH (17%)	<7.0 kg/m^2^	49%	HR 0.74 (*P* ≤ 0.01) for mortality on multivariate analysis per‐unit increase in ASMI	55 months
	W: 84		13	HCV (42%) NASH (21%) PBC (16%)	<5.5 kg/m^2^	43%	HR 0.98 (*P* = 0.92) for mortality on multivariate analysis per‐unit increase in ASMI	62 months
Nishikawa *et al*. (2017)^11^ Japan SMI by BIA	M: 205	Not awaiting LT	—	HCV (60%) Other (30%) HBV (10%)	<7.0 cm^2^/m^2^ for men	36%	HR 0.57 (*P* = 0.0005) for mortality on multivariate analysis in men per‐unit increase in SMI	3.2 years (median)
	W: 178			HCV (63%) Other (31) HBV (6%)	<5.4 cm^2^/m^2^ for women	28%	HR 0.45 (*P* = 0.0016) for mortality on multivariate analysis in women per‐unit increase in SMI	

AIH, autoimmune hepatitis; ASMI, appendicular skeletal muscle index; BIA, bioelectrical impedance analysis; CT, computed tomography; DXA, dual‐energy X‐ray absorptiometry; ETOH, alcohol; HBV, hepatitis B virus; HCV, hepatitis C virus; HR, hazard ratio; L3, third lumbar vertebrae; L4, fourth lumbar vertebrae; LT, liver transplant; M, men; MELD, model for end‐stage liver disease; NASH, non‐alcoholic steatohepatitis; PBC, primary biliary cholangitis; PSC, primary sclerosing cholangitis; PSMI, paraspinal muscle index; SMI, skeletal muscle index; W, women.

A single study (*n* = 1405, 827 men, 578 women) assessed the impact of muscle function on mortality in both sexes (*Table* 2). The Liver Frailty Index utilized a combination of grip strength, time chair stands, and balance testing to assess muscle function. Women demonstrated poorer performance across all measures when compared with men, while poorer muscle function was found to be associated with increasing mortality in both men and women.^12^

**Table 2:** Sex‐stratified association between muscle function and mortality in cirrhosis

Authors (year) Region Muscle function measure	Participants	Status	Disease severity (MELDNa)	Disease aetiology	Definition of reduced muscle function	Sarcopenia prevalence	Impact on mortality	Time to follow‐up
Lai *et al*. (2021)^12^ North America Liver Frailty Index	M: 827	Awaiting LT	18 (median)	Alcohol (33%) HCV (27%) NASH (16%)	—	—	HR 1.65 (*P* < 0.001) for mortality per 1‐SD fall in the LFI on multivariate analysis	10.2 months (median)
	W: 578		18 (median)	AIH/PBC/PSC (23%) NASH (23%) HCV (22%)	—	—	HR 1.54 (*P* < 0.001) for mortality per 1‐SD fall in the LFI on multivariate analysis	10.6 months (median)

AIH, autoimmune hepatitis; HCV, hepatitis C virus; HR, hazard ratio; LT, liver transplant; M, men; MELDNa, model for end‐stage liver disease incorporating sodium levels; NASH, non‐alcoholic steatohepatitis; PBC, primary biliary cholangitis; PSC, primary sclerosing cholangitis; SD, standard deviation; W, women.

## Discussion

This review interrogates the existing literature regarding the sex‐specific differences in muscle mass and function in cirrhosis and their relative impact on mortality for men versus women. We identified only six studies that analysed the sex‐specific impact of various measures of sarcopenia on mortality. Qualitative analysis of studies conducting sex‐stratified mortality analysis suggests that loss of muscle mass is associated with mortality in men regardless of the method used in diagnosis, but results in women are inconsistent. The single study that demonstrated a significant association between muscle function and mortality in both men and women represents an important finding that requires further validation in other cohorts.

Sex‐stratified mortality analysis of muscle mass in cirrhosis occurs infrequently in the current literature, even in those studies that collected the appropriate data. A total of 35 studies took a sex‐stratified approach to diagnosing sarcopenia in cirrhosis, but 29 of these studies failed to stratify results by sex. Only three of six studies that conducted a sex‐specific mortality analysis found a significant association between muscle mass or function and mortality in women. As women frequently make up the minority of participants in these studies, such insignificant findings may be attributed to studies being underpowered to detect the prognostic influence of sarcopenia in women. Pooling of studies is not possible due to heterogeneity of sarcopenia diagnosis, trial design, and patient population. The failure to sex‐stratify outcomes in existing cohort studies examining the mortality impact of sarcopenia in cirrhosis is a major gap in the existing literature. It reflects a lost opportunity to tease out this complex issue and means that it is not currently possible to make strong conclusion about the impact of sarcopenia in women. Since the submission of this review for publication, calls for more comprehensive research in this field have been echoed in a new addition to the literature.^13^

### Sex‐specific modulation of sarcopenia in cirrhosis

Multiple factors have been implicated in the development of sarcopenia in cirrhosis. Reduced oral intake, malabsorption, metabolic disruption, hormonal deficiency, and changes in cellular signalling result in an imbalance between muscle synthesis and breakdown.^14^ Sex‐specific factors also play a major role in the development of sarcopenia. Testosterone is a well‐recognized promoter of muscle growth through its inhibition of myostatin and promotion of insulin‐like growth factor‐1 and mammalian target of rapamycin signalling pathways in both sexes, but particularly in men.^15^ Testosterone levels fall as liver disease severity increases in men and can be reduced in up to 90% of men with cirrhosis.^16^, ^17^ Testosterone levels are also shown to correlate with muscle mass in men with cirrhosis.^18^ This may explain findings that muscle loss worsens proportionally to the severity of liver disease in men.^19^ In the geriatric population, the increased velocity of muscle loss in ageing men when compared with women is thought to be due to the greater fall in testosterone in men.^20^

There is scarce information on testosterone in women with liver disease. In a small study of 18 women with cirrhosis, testosterone levels were not significantly different to 22 healthy counterparts.^21^ A cross‐sectional study including 82 women with cirrhosis found no significant difference between testosterone levels of sarcopenic and non‐sarcopenic cohorts.^22^ Unlike in men, a comparison of liver disease severity and testosterone levels appears to be scarce in the current literature. Low free testosterone levels are associated with the development of sarcopenia in healthy women, suggesting that the influence of testosterone on muscle in women with cirrhosis still warrants exploration.^23^ The quality of available evidence is impacted using immunoassay testosterone quantitation rather than mass spectrometry; the latter technique has been found to more accurately determine the lower levels of testosterone seen in women.^24^, ^25^ The influence of oestrogens on sarcopenia is also an area of uncertainty in the field of chronic liver disease. Oestrogens are thought to have an anti‐inflammatory and anti‐catabolic effect on skeletal muscle and may even reduce muscle loss in postmenopausal women.^5^ However, the relative impact of oestrogen compared with testosterone remains controversial.^26^ Oestrogen levels are elevated in women with cirrhosis compared with healthy controls,^21^ but their overall significance and potential impact on muscle homeostasis require further investigation.

Aetiologies of liver diseases differ in their prevalence between men and women and may have unique implications for skeletal muscle. Alcohol is a direct myotoxin that decreases muscle protein synthesis and impairs the anabolic response to muscle contraction.^27^ Alcoholic cirrhosis is twice as likely to occur in men than women.^28^ It is unclear whether other specific aetiologies of liver disease impact on muscle in subjects with cirrhosis; however, there is a known association between insulin resistance and myosteatosis, as well as muscle inflammation and mitochondrial dysfunction.^29^ Given the observed insulin resistance in subjects with non‐alcoholic fatty liver disease and those infected with hepatitis C virus, these aetiologies may have a specific impact on muscle.^30^ Future large‐scale studies would benefit from both sex‐stratified and aetiology‐stratified analyses to explore the relationship between disease aetiology, sex, and sarcopenia.

### Heterogeneity in the current literature

Existing studies that report on sarcopenia in cirrhosis use a multitude of different methods to both diagnose and grade the severity of sarcopenia, most commonly using CT‐derived measures.^31^ Cross‐sectional imaging is frequently performed in people with cirrhosis during the initial transplantation assessment and for ongoing surveillance for hepatic malignancy, making it easily accessible for retrospective analysis. In this review, measurement of muscle mass by CT was predominantly cross‐sectional muscle area at L3, but other sites examined have included T12, L4, and the umbilicus. Despite the position of the umbilicus being subject to ascites and body habitus, muscle loss at this landmark has been significantly associated with mortality across several studies.^32^, ^33^ At each landmark, the choice of muscle regions to evaluate may be important when comparing muscle loss in men and women. In a comparison of SMI and psoas muscle index, psoas muscle index had limited ability to identify low muscle mass in cirrhotic men but performed reasonably well in women within the same study.^34^ Furthermore, preferential reduction of the psoas and erector spinae muscle groups has been observed in postmenopausal women while undergoing weight loss programmes.^35^ This raises the possibility that the sequence of muscle atrophy may differ according to sex, with further research required to define how best to assess muscle loss in men and women.

Expert consensus recommends that measures of muscle function be included in the diagnosis of sarcopenia, yet most studies continue to focus on estimates of muscle mass alone.^3^, ^31^ Santos *et al*. were unique among the initial 35 identified articles of this review with a sex‐specific diagnosis of sarcopenia that required both reduced function and muscle mass.^36^ However, sarcopenia by this definition demonstrated low prevalence and an insignificant impact on 1 year mortality without sex‐stratification.^36^ The definition of sarcopenia requiring composite measures of both reduced muscle mass and function has only recently appeared in the literature and requires further validation before the work performed in other medical disciplines can be paralleled.

### Potential advantages of functional muscle assessments

Reduced handgrip strength is a commonly used measure of muscle function and has predicted a significant increase in mortality for both men and women hospitalized with cirrhosis.^37^ Muscle function has also been gauged through frailty assessments including gait speed and sit‐to‐stand tests.^38^ While frailty is a uniquely important syndrome in cirrhosis, many measures of frailty evaluate muscle function, providing an important clinical intersection with the diagnosis of sarcopenia. Muscle function may indeed be a more powerful prognostic marker in cirrhosis than muscle quantity, given that muscle strength declines both earlier and more rapidly than muscle mass.^20^ Recent studies confirm this with two studies finding that handgrip strength is a better marker of prognosis than SMI in multivariate mortality analyses.^37^, ^39^ This work, in addition to the single study reported in this review finding that muscle function is prognostically relevant for both men and women, suggests that functional measures should be used in preference to estimates of muscle mass as a prognostic tool in cirrhosis.^12^

Quantitative estimates of muscle quality have been described in the current literature and may closely reflect muscle function, but no study has stratified their mortality analysis based on sex. Myosteatosis is a pathological process responsible for reduced muscle quality whereby fat accumulates within myocytes or between muscle fibres leading to muscle dysfunction.^40^ In cirrhosis, it has been suggested that increasing lipid deposition may lead to atrophy of muscle fibres and transitions of fibre type.^41^ Attenuation of skeletal muscle on CT is the most frequently adopted method of assessing myosteatosis.^42^ Several studies have observed higher prevalence of myosteatosis in women when using body mass index‐based cut‐offs, but subsequent relationships identified between survival and myosteatosis in cohorts of men and women have been inconsistent.^43^, ^44^ The prognostic value of myosteatosis in cirrhosis remains unclear, and the use of sex‐specific definitions of myosteatosis and sex‐stratified mortality analysis should be encouraged in future research.

### Prognostic application in cirrhosis

Prognostication assists in identifying patients for more aggressive intervention and is particularly important in patients with cirrhosis to better prioritize patients waitlisted for liver transplantation. The model for end‐stage liver disease (MELD) score is the most widely applied prognostic tool in hepatology. Despite its utility, evidence suggests that the MELD score may disadvantage women during prioritization.^45^ Women awaiting transplantation in America have been found to be delisted 10% more frequently than their male counterparts.^46^ Furthermore, women remaining on the transplant list may experience higher mortality due to lower functional assessment scores as compared with men.^12^ The MELD score fails to consider more recently identified markers of mortality, including sarcopenia.^47^ For this reason, many adjustments to the MELD score have been suggested in the literature, including addition of measures of sarcopenia and frailty.^48^, ^49^ Given that disparities between men and women are already recognized in transplant prioritization with the MELD score, it is even more important to understand the sex‐specific differences associated with sarcopenia. As muscle loss is more prevalent in men, the inclusion of sarcopenia into a combined prioritization score may only widen the observed gap between men and women on the waitlist. Ultimately, an accurate understanding of the prognostic value of sarcopenia and functional impairment in both men and women is imperative to ensure clinical care can be optimized for all groups.

## Conclusions

Despite varying diagnostic definitions of sarcopenia appearing in the literature, sarcopenia has been repeatedly associated with mortality in patients with cirrhosis. This review examined the sex‐specific impact of sarcopenia on mortality and found that although reduced muscle mass is invariably associated with increased mortality in men, results for women are conflicting. Reduced muscle function appears to be more prevalent in women than reduced muscle mass alone and may be a better marker of mortality risk. However, the failure to stratify mortality associations by sex in the majority of studies in this field means that it is impossible to draw strong conclusions regarding the prognostic implications of sarcopenia in women as compared with men. Given the known differences in muscle mass and function between men and women and the clear prognostic impact of sarcopenia in cirrhosis, there is an urgent need for large‐scale studies with adequate female participation and sex‐stratified analyses to better understand the diagnosis and impact of sarcopenia in both men and women with cirrhosis to improve prognostication and aid clinical management.

## Funding

None.

## Conflicts of interest

None of the authors have competing interests to disclose.

## Supporting information


**Appendix S1.** Search Strategy
