# Supplementary material for: The sex‐specific prognostic utility of sarcopenia in cirrhosis
Source: J Cachexia Sarcopenia Muscle. 2022 Aug 9;13(6):2608–15. doi: 10.1002/jcsm.13059 (PMC9745556; doi:10.1002/jcsm.13059)
Supplement: Supplementary file 1 — Appendix S1. Search Strategy [file JCSM-13-2608-s001.docx]

**Title**

The sex-specific prognostic utility of sarcopenia in cirrhosis

*Journal of Cachexia, Sarcopenia and Muscle*

**Authors**

Ryan Lowe^1,2^, Penelope Hey^1, 3^, Marie Sinclair^1, 3^

Corresponding author: Dr Ryan Lowe, Northern Health, 185 Cooper Street, Epping, Victoria, Australia. [ryan.lowe2@nh.org.au](mailto:ryan.lowe2@nh.org.au) Ph: +613 8405 8000

**Author affiliations**

1. University of Melbourne, Victoria, Australia
2. Northern Health, Victoria, Australia
3. Liver Transplant Unit, Austin Health, Victoria, Australia

**Supporting Information**

**Appendix S1. Search Strategy**

**MEDLINE**

| 1. | Liver Cirrhosis/ |
| --- | --- |
| 2. | (cirrhosis or liver fibrosis or liver failure).tw. |
| 3. | 1 or 2 |
| 4. | Frailty/ |
| 5. | Muscular Atrophy/ |
| 6. | Sarcopenia/ |
| 7. | (musc* atrophy or musc* mass or musc* wasting or musc* loss or musc* depletion).tw. |
| 8. | frail*.tw. |
| 9. | sarcopeni*.tw. |
| 10. | 4 or 5 or 6 or 7 or 8 or 9 |
| 11. | (mortality or prognos* or survival or death).tw. |
| 12. | 3 and 10 and 11 |

**Embase**

| 1. | liver cirrhosis/ |
| --- | --- |
| 2. | (cirrhosis or liver fibrosis or liver failure).tw. |
| 3. | 1 or 2 |
| 4. | frailty/ |
| 5. | muscular atrophy/ |
| 6. | sarcopenia/ |
| 7. | (musc* atrophy or musc* mass or musc* wasting or musc* loss or musc* depletion).tw. |
| 8. | frail*.tw. |
| 9. | sarcopeni*.tw. |
| 10. | 4 or 5 or 6 or 7 or 8 or 9 |
| 11. | (mortality or prognos* or survival or death).tw. |
| 12. | 3 and 10 and 11 |
| 13 | Limit 12 to (article and article in press) |
